# Semen Cassiae Extract Improves Glucose Metabolism by Promoting GlUT4 Translocation in the Skeletal Muscle of Diabetic Rats

**DOI:** 10.3389/fphar.2018.00235

**Published:** 2018-04-04

**Authors:** Meiling Zhang, Xin Li, Hangfei Liang, Huqiang Cai, Xueling Hu, Yu Bian, Lei Dong, Lili Ding, Libo Wang, Bo Yu, Yan Zhang, Yao Zhang

**Affiliations:** ^1^Key Laboratory of Myocardial Ischemia Mechanism and Treatment, Ministry of Education, Harbin Medical University, Harbin, China; ^2^Department of Cardiology, The Second Affiliated Hospital of Harbin Medical University, Harbin, China; ^3^State Province Key Laboratories of Biomedicine – Pharmaceutics of China, Key Laboratory of Cardiovascular Medicine Research, Ministry of Education, Department of Pharmacology, College of Pharmacy, Harbin Medical University, Harbin, China; ^4^Department of Medicinal Chemistry and Natural Medicine Chemistry, College of Pharmacy, Harbin Medical University, Harbin, China

**Keywords:** diabetes, Semen Cassiae, anthraquinones, anti-hyperglycaemia, PI3K–Akt–AS160–GLUT4 pathway

## Abstract

Diabetes mellitus is a clinical syndrome characterised by hyperglycaemia; its complications lead to disability and even death. Semen Cassiae is a traditional Chinese medicine, which has anti-hypertensive, anti-hyperlipidaemia, anti-oxidation, and anti-ageing properties. Our study was designed to evaluate the action of total anthraquinones of Semen Cassiae extract (SCE) on the improvement of glucose metabolism in diabetic rats and to elucidate the underlying mechanism. First, we evaluated the effect of SCE on normal rats. Next, we observed the effect of SCE using a rat model of diabetes, which was established by feeding rats with high-energy diet for 4 weeks and a single intraperitoneal injection of streptozotocin (STZ; 30 mg/kg) 3 weeks after starting the high-energy diet. Rats in different SCE groups (administered 54, 108, and 324 mg/kg/day of SCE) and metformin group (162 mg/kg/day, positive control drug) were treated with the corresponding drugs 1 week before starting high-energy diet and treatment continued for 5 weeks; meanwhile, rats in the control group were administered the same volume of sodium carboxymethyl cellulose solution (vehicle solution). One week after STZ injection, fasting blood glucose (FBG), oral glucose tolerance (OGT), fasting serum insulin (FSI) and serum lipids were quantified. Finally, the expression of proteins in the phosphatidylinositol-3-kinase (PI3K)–Akt–AS160–glucose transporter isoform 4 (GLUT4) signalling pathway was detected by western blotting. The data indicated that the levels of FBG and serum lipids were significantly lowered, and OGT and FSI were markedly increased in diabetic rats treated with SCE (108 mg/kg/day); however, SCE did not cause hypoglycaemia in normal rats. The molecular mechanisms were explored in the skeletal muscle. SCE markedly restored the decreased translocation of GLUT4 in diabetic rats. Moreover, the protein expressions of phosphorylated-AS160 (Thr642), phosphorylated-Akt (Ser473) and PI3K were significantly increased after SCE treatment in the skeletal muscle. These results indicate that SCE exerts an anti-hyperglycaemic effect by promoting GLUT4 translocation through the activation of the PI3K–Akt–AS160 signalling pathway. Our findings suggest that treatment with SCE, containing anthraquinones, could be an effective approach to enhance diabetes therapy.

## Introduction

Diabetes mellitus has become a global public health problem. In 2015, the total number of adults worldwide suffering from DM was 415 million and every 6 s a person died from DM (5 million deaths); this includes 109.6 million people in the People’s Republic of China (ranking first in the number of diabetes patients). By 2040, 642 million adults will have diabetes and the number in China will be 150 million (International Diabetes Federation, 2015). A persistently high level of BG can lead to serious complications, including damage to the hearts, blood vessels, kidneys, eyes, and nerves. DM and its complications impose an enormous burden on medical costs. This is an urgent problem for medical scientists all over the world, including China. In recent years, many clinical trials have confirmed that the positive interventions during the early period of DM to improve glucose tolerance and FBG could effectively postpone and reduce the development from prediabetes into type 2 DM (Knowler et al., 2002; DeFronzo and Abdul-Ghani, 2011; Perreault et al., 2012).

Traditional Chinese medicine is a treasure of China and an important part of traditional medicine in the world. It has a history of more than 5,000 years. DM was first recorded in *Huang Di Nei Jing*, the earliest traditional Chinese medicine records during the Warring States period to the Western Han Dynasty at more than 2,000 years ago; it was recorded as the symptom of polyuria, polydipsia, polyphagia, and weight loss and defined as “Xiao Ke Zheng” for the first time in China (Zhang, 2007; Sun, 2011). Herbs used in traditional Chinese medicine and their extracts have been reported to effectively prevent and treat DM (Liu et al., 2013, 2016; Lan and Zhu, 2015; Zhang Y. et al., 2016). Their anti-diabetic effects are multi-dimensional (Xiong et al., 1997; Li and Liu, 2010; Li et al., 2012). The combination of traditional Chinese medicine and western medicine in treating DM began in 1990s (Vray and Attali, 1995) and has become significantly popular in China (Huyen et al., 2012; Lian et al., 2015; Hu and Meng, 2017).

Semen Cassiae, called Jue Ming Zi in China, is the seed of *Cassia obtusifolia* L. or *Cassia tora* L. of the Leguminosae family initially recorded in *Shennong Bencao Jing* (Huang, 1982; Chinese Pharmocopoeia Committee, 2009). It is locally used as a variety of roasted tea. Semen Cassiae is currently used for its anti-pyretic, eyesight-improving, bowel-relaxing, anti-hypertensive, and anti-hyperlipidaemic effects (Li et al., 2003; Cheng et al., 2005; Zhang et al., 2006; Wang, 2010). It has been reported that Semen Cassiae contains anthraquinones, as the main active components, naphtopyrones, glycosides, amino acids, trace elements, polysaccharides, and other ingredients (Chunjuan et al., 2015). Fu et al. (2014) and Kim et al. (2014) found that the anthraquinones of Semen Cassiae could improve diabetic nephropathy and myocardial ischaemia and reperfusion injury in rats with DM induced by high-fat diet combined with STZ. Nevertheless, the preventive effect and underlying mechanism of SCE on DM have not yet been investigated.

The current study aimed to evaluate whether the alcohol extract (total anthraquinones) from Semen Cassiae has an anti-hyperglycaemic effect in rats with DM and to explore the possible underlying mechanisms.

## Materials and Methods

### Plant Material

For extract preparation, parched Semen Cassiae was purchased from Beijing Tongrentang (Tongrentang, Beijing, China) and authenticated by a pharmacognosy expert, Prof. Guoyu Li at Harbin University of Commerce. The plant materials of Semen Cassiae we used in this study were the seed of *C. obtusifolia* L. of the Leguminosae family. A voucher specimen (article number: 20160119) was deposited at the College of Pharmacy, Harbin Medical University, China.

### Extraction of Total Anthraquinones From Semen Cassiae

Dry Semen Cassiae (2 kg) was soaked in 50% ethanol (Lin, 2001; Liu W.M. et al., 2009; Lu and Yuan, 2010) for 12 h at a 1:7 ratio. Then it was extracted twice under heating reflux for 2 h each time. The extract was filtered and concentrated by using a rotavapor (R-300, BUCHI, Switzerland), and then lyophilised. The powder of SCE was sealed and stored in a dark at room temperature.

### Determination of Total Anthraquinones Content

#### Preparation of the Sample Solution

Semen Cassiae extract powder (0.5 g) was hydrolysed with 10% HCl (30 mL) for 20 min under ultrasonication (KQ-100VDE, Kun Shan Ultrasonic Instruments, Co., Ltd., China), and then mixed with chloroform (30 mL). The mixture was refluxed under heating for 1 h and extracted by chloroform until the chloroform layer became colourless. The combined chloroform extract was concentrated by decompression. Then, the extract was dissolved in 1% Mg(CH_3_COO)_2_ solution (50 mL), and 1 mL of the solution was diluted 100 times for detection by UV-VIS spectrophotometry (UV2550, Daojin, Japan) (Kang et al., 2011; Wang, 2013).

#### Preparation of Analytical Standards

Emodin was used as the calibration sample (Xi’an Nat Bio-technique, Co., Ltd., Xi’an, China). After accurately weighing and dissolving 1.3 mg of emodin in 1% Mg(CH_3_COO)_2_ solution, the volume was made up to 10 mL to obtain the stock solution. With 1% Mg(CH_3_COO)_2_ solution as control, the absorbance of the stock solution was scanned in the range of 400–800 nm, and the results showed that emodin maximally absorbed at 513 nm. Accurately measured 0.4, 0.6, 0.8, 1.0, 1.2, and 1.4 mL of this stock solution was added into 1% Mg(CH_3_COO)_2_ solution and the volumes were made up to 10 mL to obtain standard samples. The absorbance of different concentrations of emodin was measured at 513 nm and the standard curve was plotted. The content of total anthraquinones in SCE was calculated by interpolating with the standard curve.

### Diabetes Mellitus Animal Model

#### Animals

Male Sprague-Dawley rats (180 ± 20 g body weight) of SPF grade were purchased from Liaoning Longevity Biotechnology, Co., Ltd. (Liaoning, China). The rats were housed at controlled temperature (23 ± 2°C) and relative humidity (50–60%) and a 12 h light/dark cycle. All experimental procedures were approved by the ethics committee of the Second Affiliated Hospital of Harbin Medical University (approbation number: KY2016-226).

#### Dosage Design of SCE

The adult dose of Semen Cassiae recommended by the pharmacopoeia of the People’s Republic of China is 9–15 g per day (Chinese Pharmocopoeia Committee, 2009), and we chose a dosage of 11 g/day Semen Cassiae for the study.

As described in Section “Total Anthraquinone Content in SCE,” the content of total anthraquinones in Semen Cassiae was 6.8%. Considering the average human body weight as 70 kg, the dosage of SCE (total anthraquinones) for humans is: 11 g (weight of the original medicine of Semen Cassiae) × 6.8% (total anthraquinones content in SCE)/70 kg (average human body weight)/day = 10.69 mg/kg/day. According to the evaluation of the auxiliary hypoglycaemic function of registered health foods recommended by the China National Food and Drug Administration (China Healthy Food and Cosmetic Product Department, China State Food and Drug Administration Bureau, 2012), three doses of SCE should be tested as follows:

(1) low-dose SCE: five folds of the SCE dose for humans = 5 × 10.69 mg/kg/day (the dose of SCE for humans) = 53.45 mg/kg/day ≈ 54 mg/kg/day;
(2) middle-dose SCE: 10 folds of the SCE dose for humans = 10 × 10.69 mg/kg/day (the dose of SCE for humans) ≈ 108 mg/kg/day;
(3) high-dose SCE: 30 folds of the SCE dose for humans = 30 × 10.69 mg/kg/day (the dose of SCE for humans) ≈ 324 mg/kg/day.

#### Diabetic Rat Model

The diabetic rat model was established using a high-energy diet for 4 weeks and combined with low-dose STZ (30 mg/kg, Sigma, United States). The high-energy diet (Beijing Hua Fukang Biotechnology, Co., Ltd., Beijing, China) included lard (10.0%), sucrose (15.0%), egg yolk powder (15.0%), casein (5.0%), cholesterol (1.2%), sodium cholate (0.2%), calcium bicarbonate (0.6%), mineral meal (0.4%), and normal diet (52.6%).

#### Experimental Designing and Sampling

To evaluate the hypoglycaemic effect of SCE on normal rats, FBG of 20 male healthy Sprague-Dawley rats was quantified by a glucometer (ACCU-CHEK^®^ Active, Roche, Ireland), and then the rats were randomly divided into a control group and an SCE treatment group according to FBG with 10 rats in each group. Rats in the SCE treatment group were administered SCE at a dose of 324 mg/kg/day (SCE was dissolved in 0.5% sodium carboxymethyl cellulose solution), and the same volume (10 mL/kg) of 0.5% sodium carboxymethyl cellulose solution was administered to rats in the control group by gavage once a day for 30 days. Then, FBG levels were measured in blood samples obtained from the tip of the tail after fasting the rats for 12 h with free access to water.

To evaluate the hypoglycaemic effect of SCE on diabetic rats, FBG and BG levels after 0.5, 1 and 2 h of the administration of 2 g/kg glucose (Tianjin Xinzheng, Co., Ltd., Tianjin, China) of 80 male healthy Sprague-Dawley rats were determined after fasting for 12 h. Based on FBG and BG levels 0.5 h after glucose administration, the rats were divided into the following six groups: control group (control, *n* = 10), DM group (*n* = 14), metformin (Alphapharm, Pty, Ltd., Australia) group (MET, 162 mg/kg/day, *n* = 14), SCE-L group (54 mg/kg/day, *n* = 14), SCE-M group (108 mg/kg/day, *n* = 14), and SCE-H group (324 mg/kg/day, *n* = 14). Rats were fed the normal diet (Beijing Keao Xieli Feed, Co., Ltd., Beijing, China) for the first week of the experiment. From the second week onwards, high-energy diet was provided to rats in the DM, SCE and MET treatment groups for 4 weeks; rats in the control group were fed the normal diet. At 3 weeks, rats in all groups, except the control group, were administered STZ as a single intraperitoneal injection at a dose of 30 mg/kg. During the experimental period, SCE and MET were administered daily to the respective treatment groups by gavage. The control and DM groups were administered the same volume of vehicle solution.

#### Fasting Blood Glucose, Fasting Serum Insulin, Oral Glucose Tolerance, Area Under Curve of Glucose, and Serum Lipid Determination

One day before the experiment was completed, all rats were fasted for 12 h, and the blood samples were withdrawn from the tip of the tail to test the level of FBG. Further, rats of the treatment groups were treated with SCE or metformin, and rats in the control and DM groups were administered the same volume of vehicle solution. After 15 min, 2 g/kg of glucose was administered to the rats, and the level of BG was measured at 0.5, 1, and 2 h after glucose administration. The AUC of glucose was calculated using the following formula:

$$\begin{array}{c} {\mathrm{AUC}}_{\mathrm{glucose}}\;=\;(0hBG\;+\;0.5hBG)\times \;0.5/2 +\;(2hBG\;+\;0.5hBG)\times \;1.5/2. \end{array}$$

At the end of the experiment, the blood samples were withdrawn from the abdominal aorta after fasting for 12 h and serum samples were separated to test FSI using a rat insulin ELISA Kit (Cusabio Biotech, Co., Wuhan, China); serum levels of TC, TG, LDL-C and HDL-C were also measured by using corresponding commercially available assay kits (Sichuan Mike Biological Polytron Technologies, Inc., Chengdu, China).

### Western Blot Analysis

About 30 mg of skeletal muscle isolated from each rat was homogenised in a lysis buffer (P00138, Beyotime, Jiangsu, China) containing 1% protease inhibitor (P1005, Beyotime) and 10% phosphatase inhibitor (4906845001, Roche, United States) solution (for phosphorylated proteins) to obtain total proteins. The Membrane and Cytosol Protein Extraction Kit (P0033, Beyotime) was used to obtain membrane proteins from about 40 mg of skeletal muscle from each rat. Western blot analyses were performed using a standard blotting protocol, as described previously (Zhang Y. et al., 2016). Protein concentration was determined using the BCA protein assay kit (P0010S, Beyotime) by spectrophotometry (BioTek, United States). Total proteins (80 μg) and membrane proteins (100 μg) were separated by 10 or 8% SDS-PAGE and 8% *Bis*–*Tris* gel electrophoresis (Solarbio, China), respectively, and transferred onto nitrocellulose membranes (Invitrogen, United States). After blocking with 5% free-fat milk (BD Biosciences, United States) for 2 h at room temperature, membranes were incubated with the primary antibodies against AS160 (2670S, 1:1000, Cell Signaling Technology, Boston, MA, United States), phosphorylated-AS160 (Thr642, 4288S, 1:1000, Cell Signaling Technology), glucose transporter isoform 4 (GLUT4) (ab654, 1:1000, Abcam, Cambridge, United Kingdom), Akt (ab8805, 1:1000, Abcam), phosphorylated-Akt (Ser473, 4051S, 1:1000, Cell Signaling Technology), phosphatidylinositol-3-kinase (PI3K; 3821S, 1:1000, Cell Signaling Technology) and GAPDH (TA-08, 1:2000, ZSGB-BIO, Beijing, China) with gentle agitation at 4°C overnight and then incubated with secondary antibodies (1:10000, LICOR, United States) for 1 h at room temperature. Protein levels were quantified using the Odyssey software (LICOR) by densitometry. To normalise the data, expression levels of target proteins are presented as fold changes relative to GAPDH.

### Statistical Analysis

Data are presented as the mean ± SD. One-way ANOVA was performed for multiple comparisons analysis and two-sided Student’s *t-*test was used to compare differences between two groups by GraphPad Prism 5.0. *P* < 0.05 was considered to indicate a statistical significance.

## Results

### Total Anthraquinone Content in SCE

The standard curve was plotted with concentration as the abscissa and absorbance as the ordinate. The linear equation was *Y* = 21.324*C* + 0.024 (*R*^2^= 0.9992) with a relative standard deviation of 0.38%. The results showed that the emodin had a good linearity relationship with the absorbance in the range of 0.0052–0.0182 mg/mL. The weight of the extract obtained from 2 kg of Semen Cassiae after extraction with 50% ethanol was 334.4 g; the extraction efficiency was 16.72%. Total anthraquinones content in SCE was 6.8% (**Table 1**).

**Table 1:** Determination of total anthraquinone content in Semen Cassiae.

Sample	Total anthraquinone	Extractionrate (accounting for medicinal herbs)
Extract of Semen Cassiae by 50% ethanol	6.8%	16.72%

### Hypoglycaemic Effect of SCE

Fasting blood glucose levels in SCE-treated rats (5.3 ± 0.4 mmol/L) did not statistically differ from those in control rats (5.3 ± 0.6 mmol/L), indicating that SCE has no hypoglycaemic effect on normal rats (**Table 2**).

**Table 2:** Effect of SCE on fasting blood glucose in normal rats ($\bar{x}$ ± SD).

Groups	*n*	Dose (mg/kg)	Fasting blood glucose (mmol/L)	
			Before treatment	After treatment
Control	9	–	4.72 ± 0.37	5.33 ± 0.63
SCE	10	324	4.87 ± 0.58	5.30 ± 0.36

*Control: rats were fed with normal diet; SCE: rats were fed with normal diet and treated with SCE (Semen Cassiae extract). SCE group showed no significant difference compared with control group (*P* > 0.05).*

However, FBG levels in rats in the DM group were significantly higher than those in control rats (^∗∗^*P* < 0.01 vs. control group). The AUC of glucose in DM model rats was significantly higher than those in control rats (^∗∗^*P* < 0.01 vs. control group), indicating that OGT of DM model rats was obviously attenuated (**Table 3**). Meanwhile, FSI levels in DM model rats were lower than those in control rats (^∗^*P* < 0.05 vs. control group) and insulin resistance index (IRI) in DM model rats was higher than that in control rats (^∗^*P* < 0.05 vs. control group) (**Table 4**). The above results showed that the rat model of DM was established successfully.

**Table 3:** Effect of SCE on fasting blood glucose and glucose tolerance in diabetic rats ($\bar{x}$ ± SD, *n* = 8).

Groups	Dose (mg/kg/day)	Blood glucose (mmol/L)				AUC (mmol/L⋅h)
		FBG	0.5 h	1 h	2 h	
Control	–	5.19 ± 0.27	7.54 ± 0.53	8.44 ± 0.65	5.99 ± 0.44	13.33 ± 0.72
DM	–	20.20 ± 3.28^**^	30.40 ± 3.68^**^	29.85 ± 5.74^**^	29.10 ± 2.06^**^	57.28 ± 4.30^**^
MET	162	10.89 ± 2.35^##^	21.37 ± 5.65^##^	20.31 ± 5.65^##^	16.00 ± 8.07^##^	36.09 ± 11.12^##^
SCE-L	54	16.89 ± 2.77	29.99 ± 2.79	31.85 ± 1.33	28.14 ± 4.15	55.31 ± 4.48
SCE-M	108	11.14 ± 6.64^##^	23.48 ± 5.78^##^	29.93 ± 4.18	23.06 ± 5.08^#^	43.56 ± 9.80^##^
SCE-H	324	16.23 ± 2.91	29.49 ± 2.35	32.33 ± 1.27	26.70 ± 2.06	53.57 ± 3.09

*Control: rats were fed with normal diet. DM: diabetes mellitus, rats were fed with high-energy diet and treated with STZ. MET (metformin): DM + MET. SCE (Semen Cassiae extract): DM + SCE. L: low-dose, M: middle-dose, and H: high-dose. ^∗^*P* < 0.05, ^∗∗^*P* < 0.01 vs. control group, ^#^*P* < 0.05 and ^##^*P <* 0.01 vs. DM group.*

**Table 4:** Effect of SCE on fasting serum insulin level and insulin resistance in diabetic rats ($\bar{x}$ ± SD, *n* = 8).

Groups	Dose (mg/kg/day)	FSI (mIU/L)	IRI
Control	–	16.36 ± 2.42	3.77 ± 0.57
DM	–	13.32 ± 2.51^*^	11.65 ± 0.78^**^
MET	162	16.81 ± 5.60	7.76 ± 1.48^##^
SCE-L	54	13.91 ± 4.98	10.34 ± 3.43
SCE-M	108	18.67 ± 4.79^#^	8.28 ± 3.22^#^
SCE-H	324	17.89 ± 3.07^#^	12.73 ± 2.33

*Control: rats were fed with normal diet. DM: diabetes mellitus, rats were fed with high-energy diet and treated with STZ. MET (metformin): DM + MET. SCE (Semen Cassiae extract): DM + SCE. L: low-dose, M: middle-dose, and H: high-dose. ^∗^*P* < 0.05, ^∗∗^*P* < 0.01 vs. control group, ^#^*P* < 0.05 and ^##^*P <* 0.01 vs. DM group.*

As shown in **Table 3**, FBG levels in rats treated with SCE (108 mg/kg/day) were significantly lower than those in DM model rats (^##^*P* < 0.01 vs. DM group); moreover, the AUC of glucose was significantly lower in SCE-treated rats than in DM model rats (^##^*P* < 0.01 vs. DM group). Furthermore, as shown in **Table 4**, SCE treatment (108 mg/kg/day) significantly increased FSI levels (^#^*P* < 0.05 vs. DM group) and decreased IRI (^#^*P* < 0.05 vs. DM group). These data indicate that SCE (108 mg/kg/day) lowers FBG and IRI, thereby enhancing OGT and FSI.

### Signalling Mechanisms Responsible for the Anti-diabetic Properties of SCE in Diabetic Rats

#### SCE Promotes Glucose Uptake by Transporting GLUT4 to the Sarcolemma in Skeletal Muscle

**FIGURE 1:**
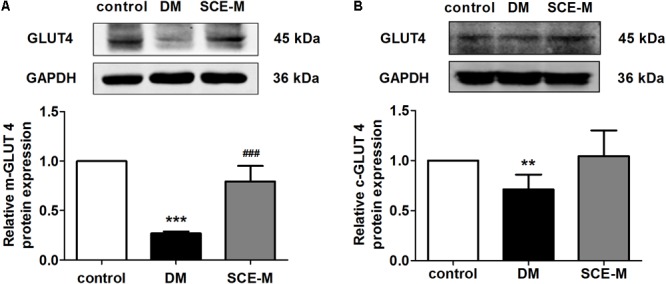
Effect of SCE on GLUT4 expression in skeletal muscle of diabetic rats. Relative protein levels of glucose transporter isoform 4 (GLUT4) in the **(A)** plasma membrane and **(B)** cytoplasm measured by western blot analysis in the control, diabetes mellitus (DM) and middle-dose of SCE-M groups (*n* = 4). ^∗∗^*P* < 0.01, ^∗∗∗^*P* < 0.001 vs. control group and ^###^*P* < 0.001 vs. DM group; mean ± SD.

After determining the hypoglycaemic efficacy of SCE, we investigated the underlying mechanism. Skeletal muscle is the major site of glucose uptake in the body (Yang, 2014). On the one hand, the skeletal muscle can store glucose as glycogen, and on the other hand, it can oxidise glucose to produce energy after glucose transfer (by glucose transporters). The major glucose transporter that mediates this uptake is GLUT4 (gene name, SLC2A4) (Huang and Czech, 2007). Therefore, we examined whether SCE could influence GLUT4 membrane trafficking. **Figures 1A,B** indicates that the protein expression of membrane GLUT4 and cytoplasm GLUT4 in the skeletal muscle of DM model rat was significantly lower than that of control rats (membrane GLUT4: ^∗∗∗^*P* < 0.001 vs. control group; cytoplasm GLUT4: ^∗∗^*P* < 0.01 vs. control group). Membrane GLUT4 expression in rats treated with SCE (108 mg/kg/day) was higher than that in DM model rats (^##^*P* < 0.01 vs. DM group, **Figure 1A**); however, the difference in the level of cytoplasm GLUT4 was not statistically significant (**Figure 1B**). This indicates that SCE (108 mg/kg/day) could promote glucose uptake by transporting GLUT4 to the sarcolemma in skeletal muscle.

#### SCE Regulates the Transport of GLUT4 to the Sarcolemma by Activating the PI3K**–**Akt**–**AS160 Signalling Pathway

In the absence of insulin stimulation, GLUT4 is stored in GLUT4 storage vesicles (GSVs). Under insulin stimulation, insulin binds to its receptor and triggers a cascade of reactions, resulting in the movement of GSVs to the cell membrane, where vesicle membranes fuse with the cell membrane, and GLUT4 is transported to the cell membrane (Zorzano et al., 1996). AS160 (Akt substrate of 160 kDa) is a substrate protein of the protein kinase Akt. Under normal conditions, its Rab-GTPase-activating protein (Rab-GAP) domain adheres to the Rab protein present on GSVs. GTPase is active at this time, and it hydrolyses GTP to GDP. Rab combines to GDP and maintains an inactive status. This makes GSVs stay in the cytoplasm. When the Akt phosphorylation site in AS160 is phosphorylated by p-Akt, Rab-GTPase is inactivated, GTP is not hydrolysed to GDP, Rab combines to GTP and maintains an active status and GSVs move to the cell membrane and transport GLUT4 to the cell membrane (Sano et al., 2003). Therefore, Akt and its downstream AS160 play an important role in GLUT4 translocation.

**FIGURE 2:**
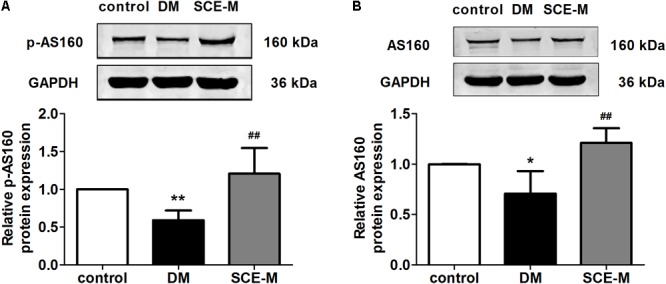
Effect of SCE on AS160 expression in skeletal muscle of diabetic rats. **(A)** Relative protein levels of the phosphorylated form of AS160 (phosphorylated at Thr642). **(B)** Total protein levels of AS160 in the control, DM and SCE-M groups (*n* = 4). ^∗^*P* < 0.05, ^∗∗^*P* < 0.01 vs. control group and ^##^*P* < 0.01 vs. DM group; mean ± SD.

**FIGURE 3:**
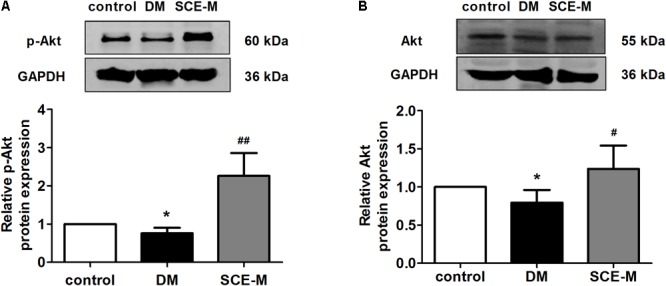
Western blot analysis of Akt protein levels. **(A)** Relative protein levels of the phosphorylated form of Akt (phosphorylated at Ser473). **(B)** Total protein levels of Akt in the control, DM and SCE-M groups (*n* = 4). ^∗^*P* < 0.05 vs. control group, ^#^*P* < 0.05 and ^##^*P* < 0.01 vs. DM group; mean ± SD.

To explore why SCE could promote GLUT4 translocation to the membrane of skeletal muscle, we determined whether SCE could influence both AS160 and Akt proteins. As shown in **Figure 2**, the protein expression of p-AS160 and t-AS160 (total AS160) was significantly lower in DM model rats than that in control rats (p-AS160: ^∗∗^*P* < 0.01; t-AS160: ^∗^*P* < 0.05 vs. control group). In contrast, levels of p-AS160 and t-AS160 expression in the SCE (108 mg/kg/day) treatment group were significantly higher than those in the DM group (^##^*P* < 0.01 vs. DM group). Similarly, the protein expression level of p-Akt and t-Akt in the DM group was significantly lower than that in the control group (^∗^*P* < 0.05 vs. control group, **Figure 3**), and levels of p-Akt and t-Akt expression were significantly higher in the SCE (108 mg/kg/day) treatment group than in the DM group (p-Akt: ^##^*P* < 0.01; t-Akt: ^#^*P* < 0.05 vs. DM group, **Figure 3**).

**FIGURE 4:**
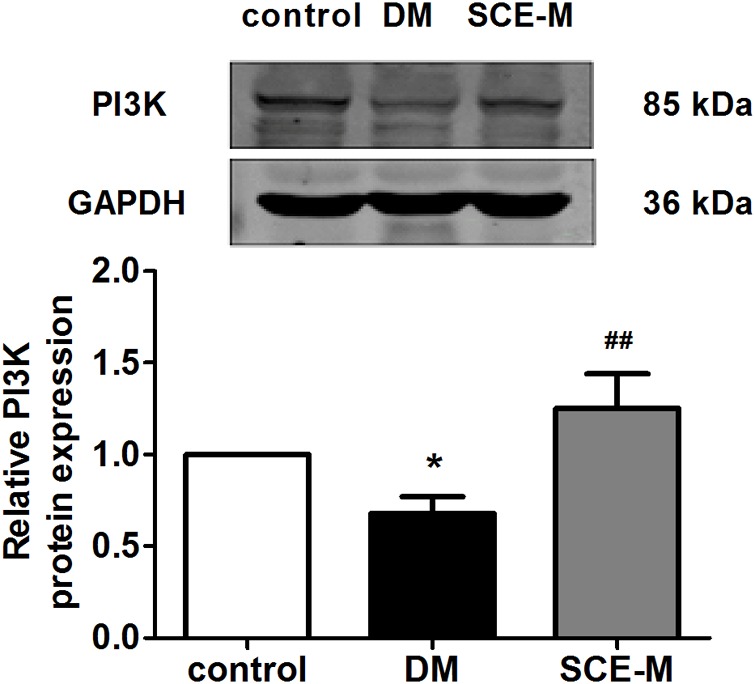
Western blot analysis of phosphatidylinositol-3-kinase (PI3K) protein levels. Relative protein levels of the PI3K in the control, DM and SCE-M groups (*n* = 4). ^∗^*P* < 0.05 vs. control group, ^#^*P* < 0.05 and ^##^*P* < 0.01 vs. DM group; mean ± SD.

Phosphatidylinositol-3-kinase activates Akt through the actions of two intermediate protein kinases, phosphosinsositide-dependent kinase 1 and Rictor/mTOR (Vanhaesebroeck and Alessi, 2000; Sarbassov et al., 2005). Thus, we evaluated the action of SCE on PI3K expression in diabetic rats. The protein expression of PI3K (**Figure 4**), upstream of Akt, was decreased in the DM group (^∗^*P* < 0.05 vs. control group), but significantly increased in the SCE (108 mg/kg/day) treatment group (^##^*P* < 0.01 vs. DM group). These data indicate that SCE could promote GLUT4 translocation by activating the PI3K**–**Akt**–**AS160 signalling pathway.

#### Hypolipidaemic Effect of SCE in Diabetic Rats

As shown in **Table 5**, levels of TC, TG, and LDL-C were all significantly higher and the level of HDL-C was significantly lower in the DM group than those in the control group (^∗∗^*P* < 0.01 vs. control group). These results indicate that diabetes-induced dysfunction in lipid metabolism was successfully induced in rats. Levels of TC and LDL-C were significantly lower and the level of HDL-C was higher in rats treated with SCE (108 mg/kg/day) than those in DM model rats (^##^*P* < 0.01 or ^#^*P* < 0.05 vs. DM group). However, there was no difference in the level of TG between the DM and SCE treatment groups.

**Table 5:** Effect of SCE on blood lipid levels in diabetic rats ($\bar{x}$ ± SD, *n* = 8).

Groups	Dose (mg/kg/day)	TC (mmol/L)	TG (mmol/L)	HDL-C (mmol/L)	LDL-C (mmol/L)
Control	–	1.33 ± 0.13	3.12 ± 0.06	0.26 ± 0.02	0.09 ± 0.02
DM	–	2.65 ± 0.35^**^	3.54 ± 0.26^**^	0.15 ± 0.02^**^	0.25 ± 0.07^**^
MET	162	2.12 ± 0.57	3.96 ± 0.56	0.29 ± 0.05^##^	0.25 ± 0.05
SCE-L	54	1.70 ± 0.34^##^	3.46 ± 0.22	0.22 ± 0.04^##^	0.20 ± 0.07
SCE-M	108	1.81 ± 0.26^##^	3.41 ± 0.16	0.20 ± 0.03^#^	0.14 ± 0.04^##^
SCE-H	324	1.82 ± 0.18^#^	3.51 ± 0.12	0.19 ± 0.02	0.19 ± 0.04

*Control: rats were fed with normal diet. DM: diabetes mellitus, rats were fed with high-energy diet and treated with STZ. MET (metformin): DM + MET. SCE (Semen Cassiae extract): DM + SCE. L: low-dose, M: middle-dose, and H: high-dose. ^∗^*P* < 0.05, ^∗∗^*P* < 0.01 vs. control group, ^#^*P* < 0.05 and ^##^*P* < 0.01 vs. DM group.*

## Discussion

**FIGURE 5:**
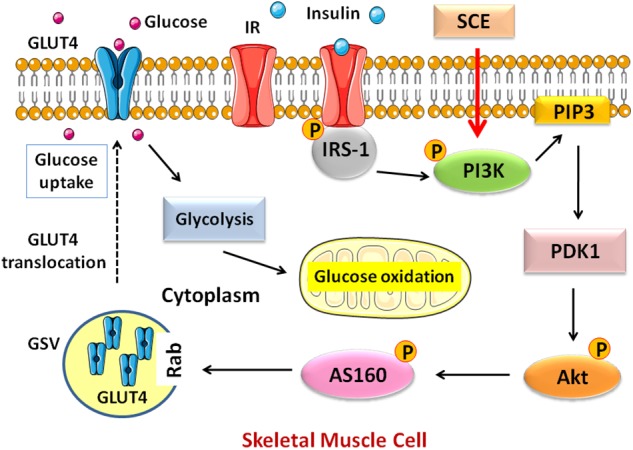
Schematic diagram of the pathway activated by SCE in skeletal muscle that regulates GLUT4 translocation to the cell membrane. GLUT4, glucose transporter isoform 4; IR, insulin receptor; IRS, insulin receptor substrate; PI3K, phosphatidylinositol-3-kinase; PIP3, phosphatidylinositol (PI)-3, 4, 5-tiphosphate; PDK1, phosphosinsositide-dependent kinase 1; Akt, protein kinase Akt or protein kinase B (PKB); AS160, Akt substrate of 160 kDa; GSV, GLUT4 storage vesicle; SCE, Semen Cassiae extract.

In the present study, we found that SCE containing anthraquinones is effective in lowering BG in diabetic rats and promoting glucose uptake by inducing the translocation of GLUT4 in skeletal muscle through the activation of the PI3K–Akt–AS160 signalling pathway. This may be the possible mechanism for anti-hyperglycaemic effect of SCE (**Figure 5**).

Type 2 DM is a metabolic disease characterised by hyperglycaemia and reduced insulin secretion either because of pancreatic β cell dysfunction or decreased insulin sensitivity (Skelly, 2006). Up to 95% of all diagnosed cases of DM in adults are of type 2 DM. Therefore, it is important to find better strategies for the treatment and prevention of type 2 DM. To achieve this goal, an appropriate experimental model is considered as an important tool for understanding the pathogenesis of type 2 DM and the effects of therapeutic agents. High-energy diet combined with STZ induces type 2 DM in rats, which simulates the human pathogenesis, and is suitable for the testing of anti-diabetic compounds (Reed et al., 2000). Zhang M. et al. (2008) demonstrated that high-energy diet combined with multiple low doses of STZ (30 mg/kg) can be used to develop a stable animal model of type 2 DM. In this study, hyperglycaemia was induced by feeding the rats with high-energy diet for 4 weeks and a single low dose of STZ (30 mg/kg) injection at the end of the third week. Rats with the FBG ≥ 7.8 mmol/L or BG level ≥ 11. mmol/L after 2 h in the OGT test were considered diabetic (American Diabetes Association, 2012). As shown in **Tables 3–5**, levels of FBG, 2-h BG, AUC of glucose, IRI, TC, TG, and LDL-C in diabetic rats were significantly increased, and FSI and HDL-C were significantly decreased, indicating that DM model rats developed glycolipid metabolism disorder and insulin resistance. Thus, the model of type 2 DM in this study was established successfully.

Drugs used in traditional Chinese medicine have shown excellent efficacy and safety in the clinical treatment of DM (Zhang Y. et al., 2008; Kianbakht et al., 2013). Semen Cassiae, a well-known traditional Chinese medicine, has been used to treat for hyperlipidaemia, DM, acute liver injury, inflammation, photophobia, hypertension, headache, dizziness, and Alzheimer’s disease (Dong et al., 2017). Management methods for agents used as both food and Chinese herbal medicines were promulgated by the (Ministry of Commerce of the People’s Republic of China, 2014), and included 101 substances as both food and medicine, including Semen Cassiae (Ministry of Commerce of the People’s Republic of China, 2014). As food, Semen Cassiae is commonly drunk as a type of roasted tea infusion for improving health in human daily life. Therefore, SCE is generally used as medicine.

To ensure the quality of research on traditional Chinese medicine, Semen Cassiae used in this study was identified by a pharmacognosy expert and they were the seeds of *C. obtusifolia* L. of the Leguminosae family. A variety of ingredients have been isolated from Semen Cassiae, and anthraquinones are considered the primary active constituents (Yang et al., 2015). Numerous studies have confirmed that the effect of alcohol extraction process used in our study is obviously better than that of water extraction process in terms of total anthraquinone extraction efficiency (Liu W.M. et al., 2009; Lu and Yuan, 2010). UV–VIS spectrophotometry is a simple, sensitive and reproducible method to quantify the content of anthraquinones in SCE (Kang et al., 2011; Wang, 2013). Our experimental data also support this, and the content of total anthraquinones extracted from Semen Cassiae was 6.8% with the extraction rate as 16.72% (**Table 1**).

Because mechanisms of compounds in the treatment of DM are very complex, the study of single Chinese medicines has become a trend to study the target and mechanism of drug action (Zhang X.N. et al., 2016). The study of Semen Cassiae in the treatment of DM and its complications is still in its initial stage. Our data (**Table 2**) showed that the administration of SCE at 324 mg/kg/day for 30 days does not affect FBG level in normal rats. Most notably, we discovered that SCE effectively lowers BG and increases insulin sensitivity in diabetic rats. It decreased levels of FBG, 2-h BG, AUC of glucose, IRI, TC and LDL-C, and increased the levels of FSI and HDL-C (**Tables 3–5**).

Skeletal muscle is the major site of postprandial insulin-dependent glucose uptake (Yang, 2014). Insulin resistance in skeletal muscle occurs much earlier before the development of β cell dysfunction and overt hyperglycaemia (DeFronzo and Tripathy, 2009). Therefore, we explored the potential mechanisms for the observed effects of SCE in skeletal muscle.

Glucose transporters (GLUTs) play an important role in regulating BG levels. GLUT4 is the most important insulin-sensitive glucose transporter protein in skeletal muscle. It is mainly localised in intracellular GSVs, and is translocated to the plasma membrane due to various stimuli to promote postprandial glucose uptake into muscle cells (Yang, 2014; Ueda-Wakagi et al., 2015). In the present study, we found that the membrane and cytoplasm GLUT4 levels in DM model rats were significantly lower than that those in control rats, and SCE (108 mg/kg/day) treatment increased the levels of membrane-bound GLUT4. Therefore, our results reveal a potential mechanism for the efficacy of SCE (108 mg/kg/day) in enhancing glucose uptake by promoting GLUT4 translocation to the cell membrane in skeletal muscle cells.

In skeletal muscle, the translocation of GLUT4 and the promotion of glucose uptake are regulated by insulin (Ueda-Wakagi et al., 2015). In the insulin pathway, the binding of insulin activates the tyrosine kinase of its receptor, which phosphorylates insulin receptor substrate-1 and activates the p85 regulatory subunit of PI3K. Activated PI3K induces the phosphorylation of downstream Akt and AS160 to regulate GLUT4 translocation. In diabetic rats, the PI3K–Akt–AS160 signalling pathway was inhibited and SCE (108 mg/kg/day) restored the activation of this signalling pathway. Therefore, SCE (108 mg/kg/day) could lower BG by regulating the PI3K–Akt–AS160–GLUT4 axis.

Previous studies have revealed that ethanol and aqueous extracts of Semen Cassiae significantly decreased the serum levels of TC, TG, and LDL-C; however, the level of HDL-C is increased (Li et al., 2002; Patil et al., 2004; Zhang et al., 2006; Wang et al., 2014). Our results show that SCE (108 mg/kg/day) significantly reduces levels of TC and LDL-C, and increases levels of HDL-C, but the levels of TG did not change compared with those in DM model rats. Our results on TG-lowering effects of SCE are different from those reported previously probably because the hypolipidaemic effect of SCE in a hyperlipidaemia model is different from that in a DM model with glucose and lipid metabolism disorder or because it may take a longer time to lower TG levels (Kim et al., 2014).

For many years, metformin has been the gold standard in the treatment of type 2 DM; therefore, we chose metformin as the positive control drug. In this experiment, metformin clearly demonstrated its hypoglycaemic effect. As shown in **Table 3**, levels of FBG, BG at 0.5, 1 and 2 h, and AUC of glucose were significantly reduced in the MET group (^##^*P* < 0.01 vs. DM group). However, there was no difference between the MET group and the DM group in levels of FSI and lipids. It makes sense, because the BG-lowering mechanism of metformin is generally attributed to decreased hepatic gluconeogenesis, delayed intestinal glucose absorption and increased glucose utilisation by the intestine, particularly anaerobic glucose metabolism (Bailey et al., 2008). This is also the reason why metformin is typically taken before meals. Therefore, Semen Cassiae is superior to metformin in regulating the level of blood lipids in type 2 DM.

The present study verifies the pharmacological effects of SCE (108 mg/kg/day), especially its effects on promoting the translocation of GLUT4 and lowing BG by promoting GLUT4 translocation through the activation of the PI3K–Akt–AS160 signalling pathway in rats with DM induced by high-energy diet and low-dose STZ. Moreover, SCE (108 mg/kg/day) exerts an anti-hyperlipidaemic effect. These are beneficial to delay the progression from prediabetes to DM. Therefore, treatment with SCE could be an effective approach to prevent the development of DM and to assist the treatment of DM. Furthermore, the PI3K signalling pathway is also commonly activated in cancer. mTOR is a major node in the pathway; approximately 50% of solid tumours are associated with the activation of the PI3K–Akt–mTOR pathway (Liu P. et al., 2009). Khan et al. (2016) found that hyperglycaemia is common in patients treated with PI3K–Akt–mTOR inhibitors; the agents targeting this pathway are associated with hyperglycaemia due to their interaction with the insulin–glucose regulatory axis. Recently, it was reported that NLRC3 is an inhibitory sensor of the PI3K–mTOR pathway in cancer (Karki et al., 2016); however, the relationship between NLRC3 and PI3K–Akt–AS160–GLUT4, NLRC3 and PI3K–Akt–mTOR signalling pathway in DM is not known. Moreover, it is not clear whether SCE can influence NLRC3 in diabetes. Besides, to deduce the exact mechanism of the anti-hyperglycaemic effects of SCE, further studies on the inhibition of the activity of GSK-3, which inhibits glycogen synthesis (MacAulay and Woodgett, 2008; Khan et al., 2017) and glucose absorption, are needed to explain the regulation of glucose homeostasis by SCE.

Numerous western medicines are derived from plants, such as artemisinin. As early as 1,578, 1,892 traditional Chinese medicines were described in *Compendium of Materia Medica* (*Bencao Gangmu*) (Cui and Li, 2006). Studies on these traditional Chinese medicines can help us discover additional drugs with high efficiencies and low toxicities. The experimental design of this study was based on a combination of ancient literature and records of traditional Chinese medicine and methods used in modern medical research. This study aims to establish standards for the quality control of plant materials and analyse active ingredients in herb formulations. Such standardized and scientific research will increase the global reach of traditional Chinese medicine.

## Conclusion

This study reveals that SCE, containing anthraquinones as the main components, prevents hyperglycaemia by promoting GLUT4 translocation through the activation of the PI3K–Akt–AS160 signalling pathway. Thus, SCE treatment could be effectively used to postpone the development of DM and to assist the treatment of DM.

## Author Contributions

YnZ and YoZ contributed conception and design of the study. MZ, XL, and BY organized the database. MZ and XL performed the statistical analysis and wrote the first draft of the manuscript. LW performed the extraction and content determination of Semen Cassiae extract. HL and HC performed the animal experiments. XH and YB performed the fasting serum insulin and serum lipids detection. MZ, HL, and LeD performed the western blot analysis. HL, HC, XH, YB, LeD, and LiD performed the FBG and BG level detection. All authors contributed to manuscript revision, read and approved the submitted version.

## Conflict of Interest Statement

The authors declare that the research was conducted in the absence of any commercial or financial relationships that could be construed as a potential conflict of interest.

## Abbreviations

AUC: area under curve
BG: blood glucose
DM: diabetes mellitus
FBG: fasting blood glucose
FSI: fasting serum insulin
GSV: GLUT4 storage vesicle
HDL-C: high-density lipoprotein cholesterol
LDL-C: low-density lipoprotein cholesterol
mTOR: mammalian target of rapamycin
OGT: oral glucose tolerance
SCE: Semen Cassiae extract
SCE-L: low-dose Semen Cassiae extract
SCE-H: high-dose Semen Cassiae extract
SCE-M: middle-dose Semen Cassiae extract
STZ: streptozotocin
TC: total cholesterol
TGs: triglycerides
